# Supplementary material for: Content-rich biological network constructed by mining PubMed abstracts
Source: BMC Bioinformatics. 2004 Oct 8;5:147. doi: 10.1186/1471-2105-5-147 (PMC528731; doi:10.1186/1471-2105-5-147)
Supplement: Additional File 2 — The original results of the above study (non-essential files are deleted to keep the file size under the limit set by BMC bioinformatics). [file 1471-2105-5-147-S2.bz2 › chilibotAdditionalFile2/dip10/622ID10417423E11008/html/CAPN2_CAPNS1.html]

 


 **CAPN2** and **CAPNS1** 
  
Found 230 abstracts in PubMed, retrieved 10.  
 

 What does Google say? 
 PDF only 
| .edu only 

---

**Interactive relationship** (e.g. stimulation, inhibition, etc)

**Neutral relationship**- 7 In order to determine which domain of the calpain I large subunit was involved in the interaction with these real clones, the 37 clones were transformed into AH109 pGBKT7 CANP  [ **CAPNS1** ]   [ **CAPN2** ]  II, AH109 pGBKT7 CANP  [ **CAPNS1** ]   [ **CAPN2** ]  III or AH109 pGBKT7 CANP  [ **CAPNS1** ]   [ **CAPN2** ]  IV.  Ref: 12358155 Hypertens Res, 2002
- Thus, we successfully constructed 4 bait expression vectors, pGBKT7 CANP  [ **CAPNS1** ]   [ **CAPN2** ] , pGBKT7 CANP  [ **CAPNS1** ]   [ **CAPN2** ]  II, pGBKT7 CANP  [ **CAPNS1** ]   [ **CAPN2** ]  III and pGBKT7 CANP  [ **CAPNS1** ]   [ **CAPN2** ]  IV, and obtained 37 real positive clones that interacted with the calpain I large subunit by screening a human heart cDNA library using pGBKT7 CANP  [ **CAPNS1** ]   [ **CAPN2** ]  as bait.  Ref: 12358155 Hypertens Res, 2002

**Non-interactive relationship** (e.g. studied together, co-existance, homology, etc.)

- it is also the major factor in the activation of the calcium activated neutral proteinase  [ **CAPNS1** ]   [ **CAPN2** ] , calpain.  Ref: 11585637 J Neuroimmunol, 2001
- The mu and m calpains consist of a mu or m 80 kDa large subunit genes Capn1 and  **Capn2** , and a common 28 kDa small subunit Capn4  [ **CAPNS1** ] .  Ref: 11602605 J Biol Chem, 2001
- In VANP and CANP  [ **CAPNS1** ]   [ **CAPN2** ] , a higher cytokine content was associated with neuropathic pain.  Ref: 12734666 Acta Neuropathol Berl , 2003
- We used immunohistochemistry including double labeling and morphometry to localize and quantify the expression of interleukin 1 beta IL 1beta, IL 6, and tumor necrosis factor alpha TNF in sural nerve biopsy samples of 41 patients with vasculitic neuropathy VANP, chronic inflammatory demyelinating neuropathy CIDP, non inflammatory chronic axonal neuropathy CANP  [ **CAPNS1** ]   [ **CAPN2** ] , and 3 controls.  Ref: 12734666 Acta Neuropathol Berl , 2003
- The selective NPR C receptor agonist, cANP  [ **CAPNS1** ]   [ **CAPN2** ]  4 23, mimicked ANF response in a dose dependent fashion.  Ref: 12829435 Am J Physiol Gastrointest Liver Physiol< MedlineTA>Am J Physiol Gastrointest Liver Physiol, 2003
- The absence of both mu and m calpain  [ **CAPN2** ]  activity, caused by disruption of the capn4  [ **CAPNS1** ]  gene in mice, retarded migration, and disrupted the cytoskeleton, both in primary capn4  [ **CAPNS1** ]  embryonic fibroblasts mEF and in capn4  [ **CAPNS1** ]  mEF immortalized with SV40 large T antigen TAg.  Ref: 12237117 Biochem Biophys Res Commun, 2002
- Cloning and molecular characterization of calpain, a calcium activated neutral proteinase  [ **CAPNS1** ]   [ **CAPN2** ] , from different strains of Schistosoma japonicum.  Ref: 11227763 Parasitol Int, 2000
- By employing this method, CANP  [ **CAPNS1** ]   [ **CAPN2** ]  activity in different regions of rat brain was determined.  Ref: 12385765 J Neurosci Methods, 2002
- The rate of gastric emptying was related to CANP  [ **CAPNS1** ]   [ **CAPN2** ]  but not gastric symptoms or actual glycemic control.  Ref: 12600185 Pol Arch Med Wewn, 2002
- 4 After the human heart cDNA library was sequentially transformed into AH109 pGBKT7 CANP  [ **CAPNS1** ]   [ **CAPN2** ] , 1000 1200 positive clones were grown on SD Trp Leu Ade His.  Ref: 12358155 Hypertens Res, 2002
- Overall cytokine immunoreactivity was highest in VANP, less strong in CIDP and lowest in CANP  [ **CAPNS1** ]   [ **CAPN2** ] .  Ref: 12734666 Acta Neuropathol Berl , 2003
- The enzyme is a heterodimer, encoded by the genes  **capn2** , for the large subunit, and capn4  [ **CAPNS1** ] , for the small subunit.  Ref: 12559913 J Mol Biol, 2003
- 3 After 4 bait vectors were transformed into AH109 by the lithium acetate mediated method, AH109 pGBKT7 CANP  [ **CAPNS1** ]   [ **CAPN2** ] , AH109 pGBKT7 CANP  [ **CAPNS1** ]   [ **CAPN2** ]  II, AH109 pGBKT7 CANP  [ **CAPNS1** ]   [ **CAPN2** ]  III, and AH109 pGBKT7 CANP  [ **CAPNS1** ]   [ **CAPN2** ]  IV were obtained, respectively.  Ref: 12358155 Hypertens Res, 2002
- The utility of the method was evaluated by assaying the proteolytic activities of trypsin and calcium activated neutral protease CANP  [ **CAPNS1** ]   [ **CAPN2** ] .  Ref: 12385765 J Neurosci Methods, 2002
